# Medicine in older age—perception and assessment of ageing processes by older people and medical and nursing professionals: study protocol for a qualitative focus group design (FOR TiMed_Life)

**DOI:** 10.1186/s12913-022-08731-7

**Published:** 2022-11-24

**Authors:** Evelyn Kleinert, Laura Mohacsi, Susanne Heim, Eva Hummers

**Affiliations:** Department of General Practice, University Medical Centre Göttingen, Georg-August-University, Humboldtallee 38, 37073 Göttingen, Germany

**Keywords:** Old age, Medical decision making, Good life, Medical and nursing professionals, Focus group discussions

## Abstract

**Background:**

This study is conducted by a subproject of the DFG research group "Medicine, Time and the Good Life" FOR 5022 (FOR TiMed_Life) and investigates the care preferences of individuals of advanced age and the care priorities of medical and nursing professionals in times of increasing medical options and more complex decision-making processes, especially for elderly patients. We assume that the preference for or rejection of medical treatment is shaped by individual and social age patterns and by the awareness of the finiteness of life. Just like older people themselves, professionals are also influenced by societal images of age(ing) and associated notions of age-appropriate health. These concepts are subject to constant change, which means that what was considered to be a 'normal' symptom of older age 100 years ago is now treated medically as a disease. The aim of the study is to identify the underlying perceptions of ageing and their influence on medical decisions.

**Methods:**

By means of semi-structured focus group discussions and supplementary individual interviews with older people and medical and nursing professionals, the subproject investigates the perception of ageing processes and the resulting care preferences and priorities of these three groups. The evaluation of the interview material is carried out in two stages: First, the recorded group discussions are pre-structured using knowledge mapping. Based on the mapping results, the interview passages are selected, transcribed, and analysed in detail based on qualitative content analysis.

**Discussion:**

Because the nature of the research question is exploratory, qualitative methods provide a suitable approach. The mapping technique provides early initial results that are used by the other subprojects. Within the research group, the results of the interviews provide an empirical basis for ethical discourses on the influence of medicine on ideas of a good life, and in particular, on successful ageing.

**Trial registration:**

German Clinical Trials Register: DRKS00027076, 05/11/2021.

## Background

As a result of demographic changes,’old age’ is becoming increasingly important from both an individual and a societal perspective. On an individual level, the significant increase in life expectancy, which continues to rise, means an extended phase of life in old age [1]. In Germany, life expectancy for those born in 2019 is 79 years for men and 83 years for women. This means that it has almost doubled since 1900 [2]. Parallel to the increase in life expectancy that is due to better living conditions and medical care, the possibilities offered by modern medicine have led to a change in the perception of the ageing processes in recent decades. What was long regarded as a 'normal' sign of ageing, appropriate to the stage of life, is increasingly seen as a disease, and as such is monitored by doctors and treated with drugs or surgery. In this process, age-related norms and expectations are changing, and a discrepance between trivialisation and pathologisation of ageing processes arises in everyday practice [3]. These shifts in perceptions also include a positive change in images of old age within the past 25 years. Previously dominant views of decline (in terms of loss of vitality, resilience, and social contacts) have been replaced by more resource-oriented views (opportunities for further development and planning of one's own life). People with higher educational qualifications benefit more from these opportunities than less educated people [4, 5], however. Increasing life expectancy and a higher level of prosperity have resulted in a social change of old age as a stage of life. This led to the concept of the so-called ‘new elderly’, who still have many years to live after reaching the seventh decade and whose health and socioeconomic situation allows them to organise these years according to their own interests and to realise some individual ambitions [6]. As ageing perceptions have changed, health-related behaviours (e.g., physical activity or use of preventive services) have also changed, but this again depends on the education level and in some cases, on sex [7]. Age perceptions and health-related behaviour are interdependent in the sense that people with negatively connotated age perceptions do less for their health than people with positive age perceptions. This provides an important addition to the classic health behaviour models and the consistently reported influence of socioeconomic factors on health-related behaviours [8]. In addition, control beliefs and a more positive view of ageing are related. This means that people who believe in the impact of their behaviour on their health and living conditions tend to focus on the benefits of ageing and its gains in life experience [1].

The causal attribution of symptoms has a major impact on healthcare behaviour as well. Various studies show that people who attribute health problems to their age are less likely to seek medical help than those who consider their complaints to be related to illness [9, 10]. However, these studies do not explain the influence that causal attribution or the individual's own image of old age might have on the patient's treatment priorities. Furthermore, the perspectives of medical and nursing staff have hardly been examined to date, although they are involved in two respects: 1) they have to deal with the healthcare priorities of the patients, and 2) they influence both the healthcare priorities and the age images of the patients by trivialising or pathologising certain symptoms. There are indications that the images of age held by medical and nursing professionals have not changed to a similar extent because medical developments are still stereotyped [11]. Medical care for the elderly, who often have several health conditions (multimorbidity), differs from care for younger patients. In the context of multimorbidity, not every generally indicated therapy with its respective risks and side effects is reasonable and might do more harm than good [12–15]. A geriatric general medical treatment focus therefore has the goal of preserving the autonomy and quality of life of older people as much as possible, and also tries to avoid or reduce the need for long-term care. According to this approach, priority should be given to the treatment of those diseases whose therapies contribute most to maintaining activity and quality of life, in order to limit undesirable side effects, interactions, and risks [13]. Therefore, the so-called functional age of patients is considered regarding treatment decisions (in distinction to the calendrical age) [16–18]. Empiric surveys also suggest that including age in decision making in the direct physician–patient relationship is deemed appropriate [19–21].

This case-related decision making in the context of a geriatric treatment focus differs fundamentally from economically oriented discourses about a general prioritisation or even rationing of access to medically indicated procedures. These discourses have been shaped by the expansion of medical services (for all age groups) combined with limited financial resources of the welfare state since the 1990s [22]. Although there is no explicit age-related rationing of medical resources in Germany, implicit rationing does take place in the context of individual treatment decisions, which leads to great uncertainty and stress in physicians and can affect the relationship with patients [23–25]. In light of the current COVID-19 pandemic and the associated discussion about the possible need to prioritise scarce intensive-care capacities, this problem has taken on new relevance. Although the relevant recommendations of various professional societies identify the clinical prospects of success of intensive-care treatment as decisive criteria for prioritisation [26], these prospects of success are repeatedly mixed in the public and political discussion with barely reflected ideas about the usefulness of intensive-care treatment in old age [27]. The care preferences and priorities to be explored in this subproject can also contribute to illuminating the preconditions of these prioritisation discourses, but this aspect is not the focus of this review.

The ethical concept of futility refers to assumptions about the success of life-sustaining or life-saving treatment that is considered futile because of specific conditions and therefore cannot be in the patient's best interest. Nevertheless, the futility concept is associated with widely varying conjectures about quality and duration of life. These conjectures depend on the (individual?) perspective and cannot be ethically justified [28]. It is of limited use in clinical decision making because it places high demands on the involved parties and can lead to conflicts between professionals, patients, and relatives when views on appropriate quality of life differ [28, 29].

In qualitative interviews with U.S. war veterans and their primary care physicians, Rodriguez and Young discovered that four factors were relevant to both groups regarding end-of-life medical treatment: impact on the quality of life; emotional, financial, and other costs; likelihood of success; and impact on longevity. Patients had the impression, however, that their physicians attributed greater relevance to the impact on lifespan than to the other factors, whereas for themselves, considerations of past, present, and future quality of life were decisive in determining which treatments they considered acceptable. From the perspective of a physician, decisions between quality of life and guideline-appropriate treatment of physical conditions were particularly conflicting [30]. Different perspectives of patients and professionals on health are also reflected in the discrepancy between so-called objective (medical diagnoses) and subjective health assessment [7]. Surveys on the subjective health status conducted as part of the German Ageing Survey (DEAS) showed that subjective health decreases with ageing, but it does so to a lesser extent than objective health status [5]. In the DEAS survey of 2014, more than half of the multimorbid persons described their subjective health as good [7]. These coping processes have also been demonstrated in various psychological studies [31, 32]. It can be assumed that the subjective evaluation of one's own state of health is influenced by age-related norms regarding a good life.

However, the current research situation does not explain to which extent the elderly have an 'appropriate' acceptance of their age and health situation, which might also include a sense of peace at the thought of dying. These might also be effects of unnecessarily resigned images of old age, in case medical treatment could still make an improvement in the patient's situation. In this respect, elderly people may not only experience overtreatment due to unnecessary, futile, or even harmful therapies, but also to undertreatment in some cases.

The Sixth Report of the Federal Ministry for Family Affairs, Senior Citizens, Women and Youth (BMFSFJ) on the situation of the older generation in Germany indicates an insufficient supply of psychotherapeutic and psychiatric care, especially the treatment of dementia and depression in old age, due to prejudicial images of old age. There is a widespread assumption, not only among patients, but also among professionals, that mental adaptability (plasticity) declines with advancing age. In addition, people with cerebral diseases, for example, are perceived as 'difficult patients'. Both age images can result in a lack of provision or utilisation of appropriate psychotherapeutic and psychiatric treatments [22]. Whereas single studies exist on the physician's perspective on the appropriateness of medical treatments, the nursing perspective has hardly been investigated so far. The reason might be that nurses do not make any direct therapeutic decisions. On the other hand, they execute the physician's decisions in nursing care and thus also have a steering effect within the boundaries of their operational scope. In addition, they often have a more direct experience of the consequences of medical decisions for patients because they spend much more time with them.

It is known from Nursing Sciences that the nurses' job satisfaction decreases and their emotional exhaustion increases when they have to perform futile treatments or are in charge of caring for patients treated in this way [33, 34]. Even though the patient's will has been increasingly addressed since the 1960s in the context of shared decision making and has also been the subject of scientific research [35], normative concepts of the good life and their influence on health care have not been considered in health care research to date. The concept of quality of life alone provides indications in this respect. Considering this, the question arises how physicians can integrate the subjective concepts of good life into their treatment decisions without risking over- or undertreatment. On the basis of previous research results, it is not possible to derive what might be good and reasonable concepts for the treatment of medical conditions in old age with regard to both patients and professionals.

### The aim of the study

The aim of this subproject is to investigate the care preferences of older people and the care priorities of medical and nursing professionals in relation to normative images of old age and attitudes towards the finiteness of life. Against the background of an ageing society and a continuous increase in medical possibilities, this subproject aims to provide guidance on how medical progress can be used in a meaningful way, considering ideas of a 'good life' and recognising the importance of the duration of life and its finiteness. The empirical data are meant to provide a differential view of perspectives of various actors in the healthcare system and to support an ethically reflected, patient-centred care of elderly, multimorbid patients.

## Methods

### Design

In this study, methods from qualitative social research are used, specifically, focus group discussions with medical and nursing staff entrusted with the care of the elderly. Focus group discussions and supplementary individual interviews with people age 75 and older complete the survey.

### Focus group discussions

In qualitative social research, focus groups (instead of individual interviews) are used to gain an overview of the range in variation and structure of opinions on a particular topic, with a lower investment of personnel and time [36]. Interaction and communication within the group has the advantage that the participants inspire each other with their statements. In this way, topics can be explored much more widely, diversely, and in some cases, more creatively than in individual interviews. The guidelines for the focus group discussions will be developed in the working group in consultation with another subproject (Ethics of Geriatric Medicine). For each stakeholder (elderly, physicians, and nursing professionals), the guideline will be tested in one pilot focus group and adapted if necessary. The discussions will be audio-recorded and explained in the information letter, and all participants will sign an informed consent form in advance. Four group discussions with 8 persons each are planned: four groups with persons of advanced age (seniors over 75 years), four groups with physicians, and four groups with nursing staff. In total, the perspectives of 96 people in 12 group discussions will be included in the analysis. Each group discussion will take 1.5 to 2 h.

### Individual interviews

In order to complement the findings over and above the focus groups, supplemental guided interviews are planned with selected individuals over the age of 75 (approximately 10 persons, depending on saturation). For this purpose, persons will be selected who are unable or unwilling to participate in a group discussion for health or personal reasons. For these face-to-face interviews, the guideline for the focus groups is modified on the basis of the initial evaluations. Duration of the interviews depends on the interviewees' health capabilities and can vary between 30 and 90 min.

### Recruitment/sampling

The study will focus on **old and very old people** (from 75 years of age; further referred to as seniors), physicians who predominantly care for old people on outpatient or inpatient basis, as well as nursing staff from outpatient and inpatient geriatric care, and from geriatric or palliative wards in hospitals or in hospices. In order to represent the diversity of socio-cultural characteristics of these three groups, the selection of respondents will consider their sex, educational level, and cultural background. Due to the increasing number of semi-skilled assistants in elder care, partly with a low scope of employment, the educational level and the average weekly working hours of the caregivers are also considered in the sampling. We intend to include a variety of professional backgrounds. In order to reflect a wide range of perspectives among the seniors, the survey includes the current state of health in addition to sociodemographic factors. For this purpose, two global questions of the SF-12, an abbreviated version of the General Health Status Questionnaire (SF-36), are used [37].

Medical and nursing staff are recruited via existing networks of the Institute of General Medicine (mail distribution list) and in a direct approach of clinics and facilities of outpatient and inpatient geriatric care. To recruit the seniors, advertisements are placed in the local daily press. Visitors to community or senior centres or to educational, sports, or cultural programs for seniors are addressed through flyers, posters, and project presentations. Those interested in participating in a focus group discussion or individual interview will receive the study information and reply forms with the informed consent forms.

### Data analysis

The analysis of the focus group discussions will identify central statements and structure them systematically. The focus of the analysis is not on individual contributions to the discussion, but on presenting the spectrum of opinions of the entire group [38].

The method of knowledge mapping is used for the efficient qualitative assessment of the large, hardly structured data volumes that arise. For this purpose, the recorded discussion is gradually and systematically evaluated by the project team. Using the card technique, a logical condensation and classification of the spoken material is generated [39]. Interim analyses will be conducted after the first and second focus group discussions of each group of participants. These first results will be used as inputs for the further development and adaptation of the focus group and interview guidelines. In addition, they provide an opportunity for the subprojects to exchange information with each other. During the second step, a group-wide synthesis on specific topics will be developed. This will be used as foundation for a deeper content analysis after the data collection is completed. For this purpose, relevant parts of the group discussions as well as the entire individual interviews will be transcribed verbatim and analysed using qualitative data analysis software (MaxQDA®). Based on the content analysis according to Kuckartz, inductive and deductive categories are defined and compared with the results of the previous analysis step and adjusted if necessary [40]. The categories already generated by the mapping process serve as initial categories that are validated and expanded by the in-depth analysis.

### Data management

In addition to the audio recordings of the focus group discussions and the individual interviews, the following personal data will be collected from all participants for the contacting and scheduling of appointments: Last name, first name, title (if applicable), e-mail, and telephone number. In order to be able to include the different life situations and social or professional backgrounds of the patients, the following characteristics are also recorded in the sampling process (Table 1).

**Table 1:** Considered characteristics of the participants

Seniors	Physicians	Nurses
- Age - Sex - Migration background - Level of education - General state of health (two global questions of the SF 12)	- Age - Sex - Migration background - Years of professional experience - Specialist examination (year/subject) - Current occupation (specialty; outpatient vs. inpatient)	- Age - Sex - Migration background - Years of work experience - Qualification - Area of current activity - Weekly working hours

### Inclusion and exclusion criteria

#### General inclusion criteria (apply to all three groups):

- Sufficient knowledge of German
- Interest in the research question
- Willingness to participate in the study and consent to audio recording of the discussions/conversations

#### Specific inclusion criteria for physicians:

- Professional activity in medical care of the elderly

#### Specific inclusion criteria for nurses:

- Professional activity in outpatient or inpatient geriatric care or in geriatric or palliative care wards in hospitals or in a hospice

#### Specific inclusion criteria for seniors:

- Age ≥ 75 years
- Sufficient hearing ability
- Cognitive and social ability to actively participate in a group discussion or alternatively, in an individual interview

#### General exclusion criteria (apply to all three groups):

- Lack of command of German
- Lack of cognitive and social ability to participate in a structured group discussion

### Drop-out criteria and withdrawal

Data collection or analysis will be stopped as soon as a participating subject withdraws their consent to study participation. Since this study is a survey and no intervention takes place, no further drop-out criteria are necessary.

### Legal and ethical aspects

The study will be conducted in accordance with the Declaration of Helsinki in its current version and with the principles of good research practice. The data collection is based on the legal requirements of the European Union (DSGVO), Germany (BDSG), and the State of Lower Saxony.

The study protocol has been approved by the Ethics Committee of the University Medical Centre Göttingen before the start of the study (Ethics Approval No. 16/9/21). Participation in the study is voluntary, and consent may be withdrawn at any time without giving any reason and without detriment to medical care or the workplace. When informed consent for focus group participation is withdrawn, the data that have already been collected will be anonymised immediately and will be used in this form from then on. When informed consent for an individual interview is withdrawn, the data that have already been collected will be deleted, or the participant will be asked whether the already existing material may be evaluated further. Personal data will be deleted immediately in the event of withdrawal, regardless of whether participation was in a focus group or individual interview.

Participants will be informed in written form before the start of the study about the nature and scope of the planned research, in particular about the possible benefits and risks for their health. Participants have the opportunity to ask questions in person. Consent is documented by signature on the consent form.

### Risk–benefit analysis

Since this study will survey three groups of people, these groups will have different benefit-risk profiles. The group of seniors is the most vulnerable group and might be psychologically burdened by the discussion of health crisis situations. However, this possible distress is likely to be rather minor and temporary. In addition, experience shows that a focused engagement with important aspects of life is also a part of mental well-being. Study participation does not affect the medical care of participants. In addition, the self-determination of the participants is guaranteed for the entire study period. Participants can drop out of the study at any time.

The nurses and physicians participate as experts in the focus group discussions. They are not expected to find study participation particularly stressful. It is ensured that no direct colleagues are in the same group, so that personal attitudes of the participants do not become unintentionally known in their professional contexts. Participants are advised that the discussed contents must be treated confidentially.

### Archiving and data protection

The personal data collected within the research project after informed consent of the participants as well as the audio data from the group and individual interviews are subject to the duty of confidentiality and the abovementioned data protection regulations. They are encoded (pseudonymised) and password-protected on the servers of the Department of General Practice. Only authorised project staff of the Department of General Practice of the University Medical Centre Göttingen and the Department of Ethics in Medicine of the Carl von Ossietzky University Oldenburg have access to these original data. All project staff are required to maintain confidentiality. The transfer of these data between the two institutes is encoded via Cryptshare.

In addition to the names of persons and institutions, places and other clearly identifying contents are also alienated in the process of transcription. The identification of participants thus becomes impossible or would involve a disproportionately large effort (de facto anonymisation). Transcription is performed at the Department of General Practice using the transcription software 4fx, which is DSGVO-compliant and uses its own servers in Germany. This means that only authorised project staff members have access to the original data.

Data without personal reference or (de facto) anonymised data are shared with the entire DFG research group via a cloud infrastructure of the Göttingen-based Society for Scientific Data Processing (Gesellschaft für wissenschaftliche Datenverarbeitung mbH Göttingen, GWDG). The DFG research group uses the data in (de facto) anonymised form. The publication of study results in scientific journals and at scientific congresses is strictly anonymous.

The audio recordings are deleted after the end of the evaluations, as are the participants' contact data, including the pseudonymisation keys. The (de facto) anonymised transcripts and contextual information will be archived password-protected at the Institute of General Practice for a period of 10 years beyond project duration.

## Discussion

The aim of the study is to investigate the care preferences of older people and the care priorities of medical and nursing professionals in relation to normative perceptions of old age and attitudes towards the finiteness of life. The research question requires an open, hypothesis-generating methodological approach. Because individual interviews take a relatively large number of resources, we mainly use focus group discussions with the three groups and only supplementary individual interviews with older participants with health problems.

As one of seven subprojects of the interdisciplinary DFG research group "Medicine and the Time Structure of Good Life", we aim to disseminate our findings to the other subprojects as early as possible. We address this issue by using the method of knowledge mapping prior to the evaluation by means of qualitative content analysis. Thus, this subproject widens the ethical discourses on ideas of good life in relation to older age as well as to a medical and nursing perspective and contributes significantly to medical ethical theory building.

## Data Availability

Not applicable.

## Abbreviations

DFG: Deutsche Forschungsgemeinschaft (German Research Foundation)
DSGVO: Datenschutz-Grundverordnung (General Data Protection Regulation)
BDSG: Bundesdatenschutzgesetz (Federal Data Protection Act)
GWDG: Gesellschaft für wissenschaftliche Datenverarbeitung mbH Göttingen (Goettingen-based Society for Scientific Data Processing)
